# A deep learning model for gastric diffuse-type adenocarcinoma classification in whole slide images

**DOI:** 10.1038/s41598-021-99940-3

**Published:** 2021-10-14

**Authors:** Fahdi Kanavati, Masayuki Tsuneki

**Affiliations:** 1 Medmain Research, Medmain Inc., Fukuoka, 810-0042 Japan; 2 Medmain Inc., Fukuoka, 810-0042 Japan

**Keywords:** Gastrointestinal cancer, Classification and taxonomy, Machine learning

## Abstract

Gastric diffuse-type adenocarcinoma represents a disproportionately high percentage of cases of gastric cancers occurring in the young, and its relative incidence seems to be on the rise. Usually it affects the body of the stomach, and it presents shorter duration and worse prognosis compared with the differentiated (intestinal) type adenocarcinoma. The main difficulty encountered in the differential diagnosis of gastric adenocarcinomas occurs with the diffuse-type. As the cancer cells of diffuse-type adenocarcinoma are often single and inconspicuous in a background desmoplaia and inflammation, it can often be mistaken for a wide variety of non-neoplastic lesions including gastritis or reactive endothelial cells seen in granulation tissue. In this study we trained deep learning models to classify gastric diffuse-type adenocarcinoma from WSIs. We evaluated the models on five test sets obtained from distinct sources, achieving receiver operator curve (ROC) area under the curves (AUCs) in the range of 0.95–0.99. The highly promising results demonstrate the potential of AI-based computational pathology for aiding pathologists in their diagnostic workflow system.

## Introduction

According to the global cancer statistics 2020^1^, gastric cancer is amongst the most common leading causes of cancer related deaths in the world which is estimated 769,000 deaths and ranked fifth for incidence and fourth for mortality globally. Symptoms of gastric carcinoma tend to manifest only when it is at an advanced stage. The first sign is the detection of nodal, hepatic, and pulmonary metastases. In countries with a high incidence of gastric cancer, especially Japan, the increased use of endoscopic biopsy and cytology has resulted in the identification of early stage cases which has resulted in an increase in survival rates^2–5^. Microscopically, nearly all gastric carcinomas are of the adenocarcinoma (ADC) type and are composed of foveolar, mucopeptic, intestinal columnar, and goblet cell types^6^. According to the Lauren classification^7^ gastric ADCs are separated into intestinal and diffuse types. The intestinal-type shows well-defined glandular structures with papillae, tubules, or even solid areas. By contrast, the diffuse-type consists of poorly-differentiated type and signet ring cell carcinoma (SRCC). Diffuse-type ADC scatters and infiltrates widely, and its cells are small, uniform, and cohesive. Often these cells exhibit an SRCC appearance with the intracytoplasmic mucin pushing the nucleus of the neoplastic cells to the periphery. The amount of mucin present in these cells may be highly variable and difficult to appreciate in diffuse-type ADCs. Diffuse-type ADCs are more challenging to diagnose than other gastric carcinomas such as the intestinal-type. Diffuse-type cells are often single and inconspicuous in a background desmoplasia and inflammation, and they can often be mistaken for a variety of non-neoplastic lesions including gastritis or reactive endothelial cells in granulation tissues. Surgical pathologists are always on the lookout for signs of diffuse-type gastric adenocarcinoma when evaluating gastric biopsies.

Deep learning has found many successful applications in computational pathology in the past few years for tasks such as tumour and mutation classification, cell segmentation, and outcome prediction for a variety of organs and diseases^8–21^. For stomach in particular, Sharma et al.^22^ trained a model for carcinoma classification using a small training set of 11 WSIs, while Iizuka et al.^21^ trained a deep learning model using a large dataset of 4,036 WSIs to classify gastric biopsy specimens into adenocarcinoma, adenoma, and non-neoplastic.

In this paper, we trained deep learning models for the classification of diffuse-type ADC in endoscopic biopsy specimen whole slide images (WSIs). To do so, we used two approaches: one-stage and two-stage. With the one-stage approach, the model was trained to directly classify diffuse-type ADC. With the two-stage approach, we used the model of Iizuka et al.^21^ to first detect ADC, followed by a second stage model that subclassifies the detected ADC cases into diffuse-type ADC vs other ADC. For both approaches, we have used the partial transfer learning method^23^ to fine-tune the models. We obtained models with ROC AUCs in the range in 0.95–0.99 for the five independent test sets, demonstrating the potential of such methods for aiding pathologists in their workflows.

## Results

**Figure 1:**
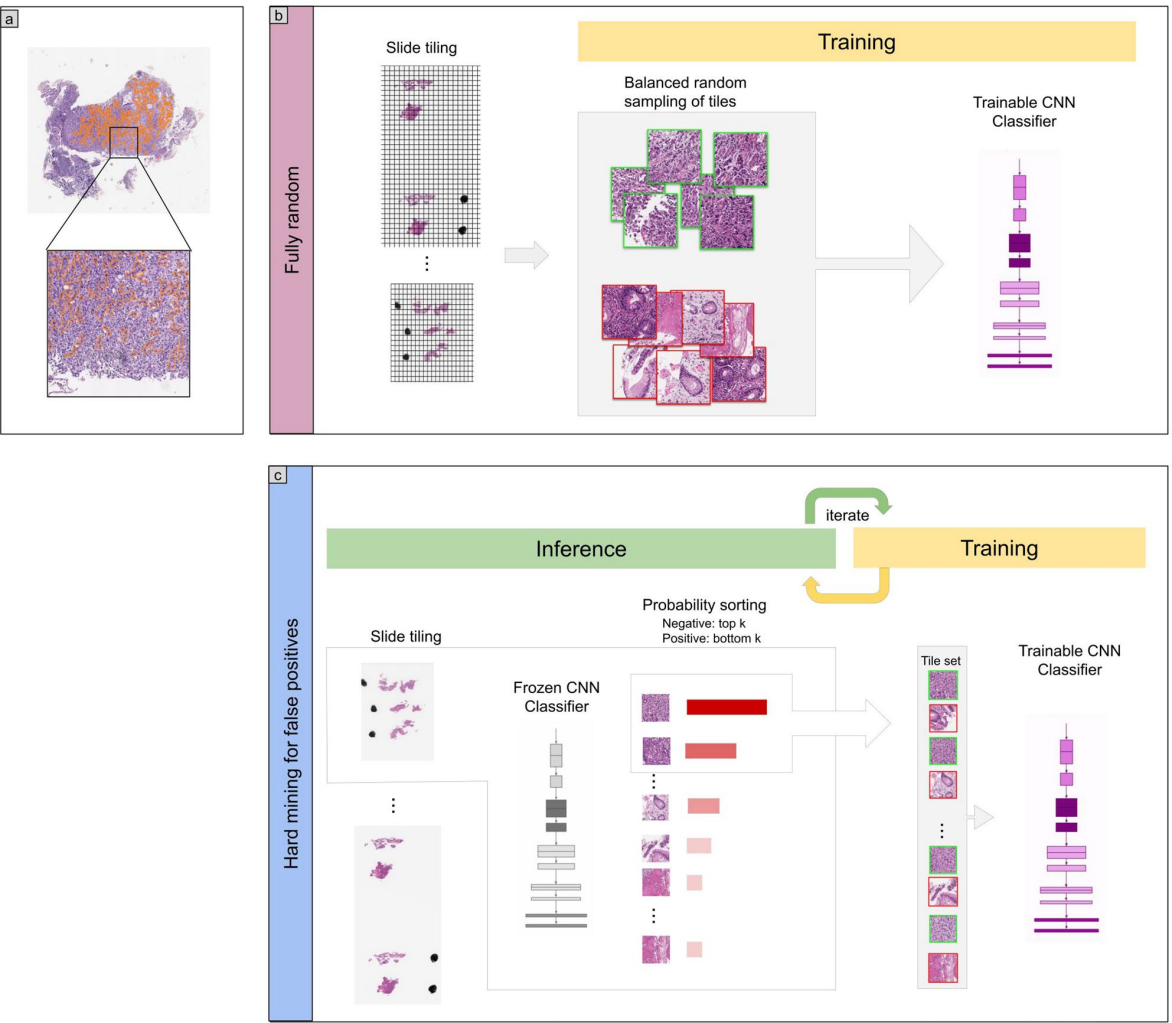
Method overview. (**a**) An example of diffuse-type ADC annotation that was carried out digitally on the WSIs by pathologists. (**b**) The initial training consisted in fully-random balanced sampling of positive (diffuse-type ADC) and negative tiles to fine-tune the model. (**c**) Once there was no further improvement on the validation set after 2 epochs, the training switched into hard mining of tiles, which is an iterative process that alternates between training and inference. During the inference step, we applied the model in a sliding window fashion on all of the WSI and selected the k tiles with the highest probabilities if the WSI was negative, and k tiles with the lowest probabilities if the WSI was positive. The tiles were collected in a subset, and once the subset reached a given size, it was batched and used for training. This process allows training on the hardest examples and reduce false positives.

The aim of this study was to train a convolutional neural network (CNN) for the classification of diffuse-type ADC in biopsy WSIs. In order to apply a CNN on the large WSIs, we followed the commonly adopted approach of tiling the WSIs by extracting fixed-sized tiles over all the detected tissue regions (see methods section for more details). Overall, we trained four different models: (1) a two-stage method using existing model of Iizuka et al.^21^ to first detect ADC, followed by a second model that detects diffuse-type ADC, both at $\times$ 10 magnification; (2) a one-stage method for direct diffuse-type ADC classification at magnification $\times$ 10 and a tile size of 224 $\times$ 224 px; (3) a one-stage method for direct diffuse-type ADC classification at magnification $\times$ 20 and a tile size of 224 $\times$ 224 px; and (4) a one-stage method for direct diffuse-type ADC classification at magnification $\times$ 20 and a tile size of 512 $\times$ 512 px. Figure 1 provides an overview of the training of a given model. At $\times$ 10 magnification 1 pixel corresponds to $1\,\,{\upmu }\hbox {m}$, and at $\times$ 20, 1 pixel corresponds to $0.5\,\,{\upmu }\hbox {m}$.

### Evaluation on five independent test sets from different sources

**Figure 2:**
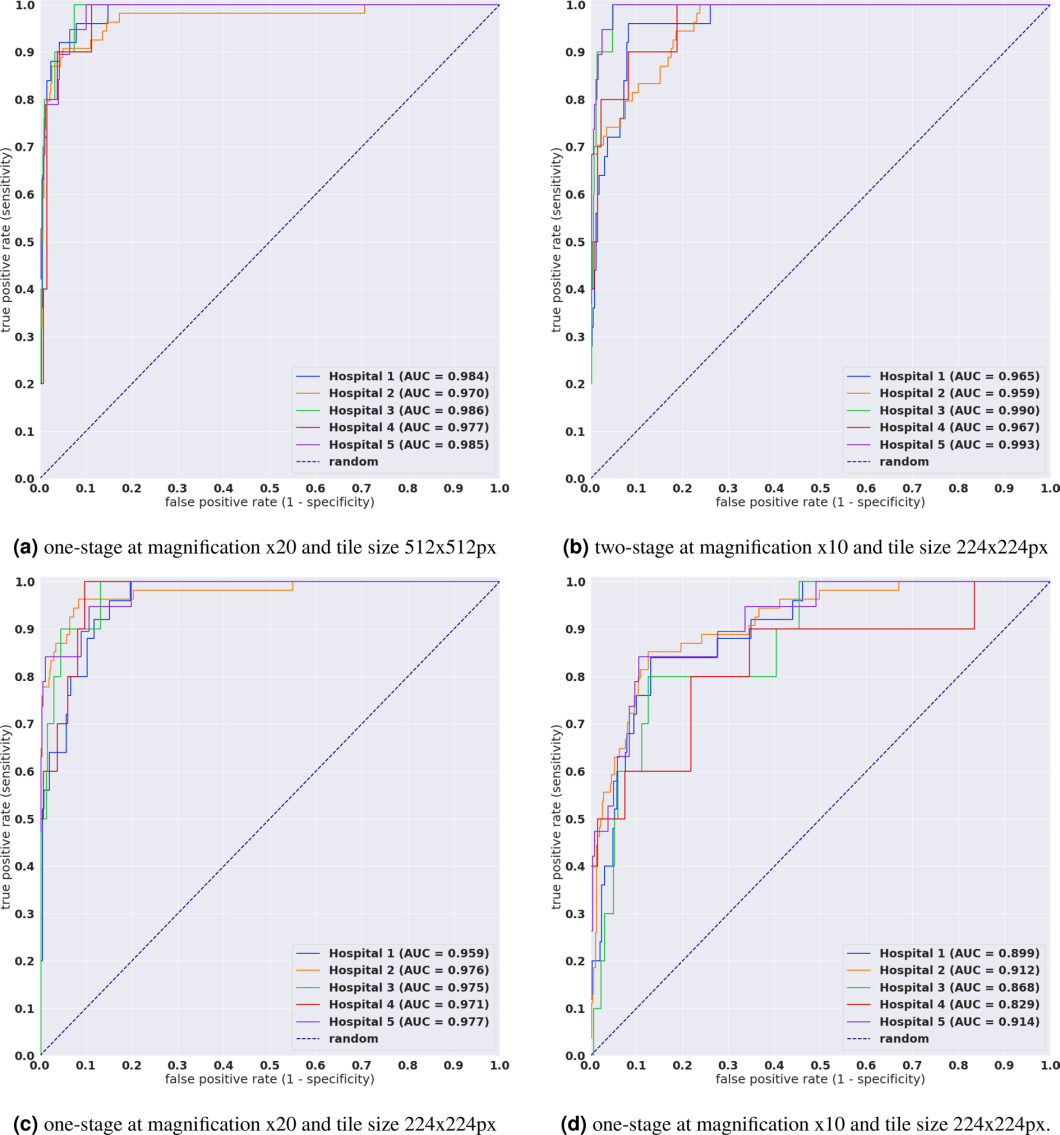
ROC curves and corresponding AUCs for the test sets from five different hospitals using four different methods.

**Figure 3:**
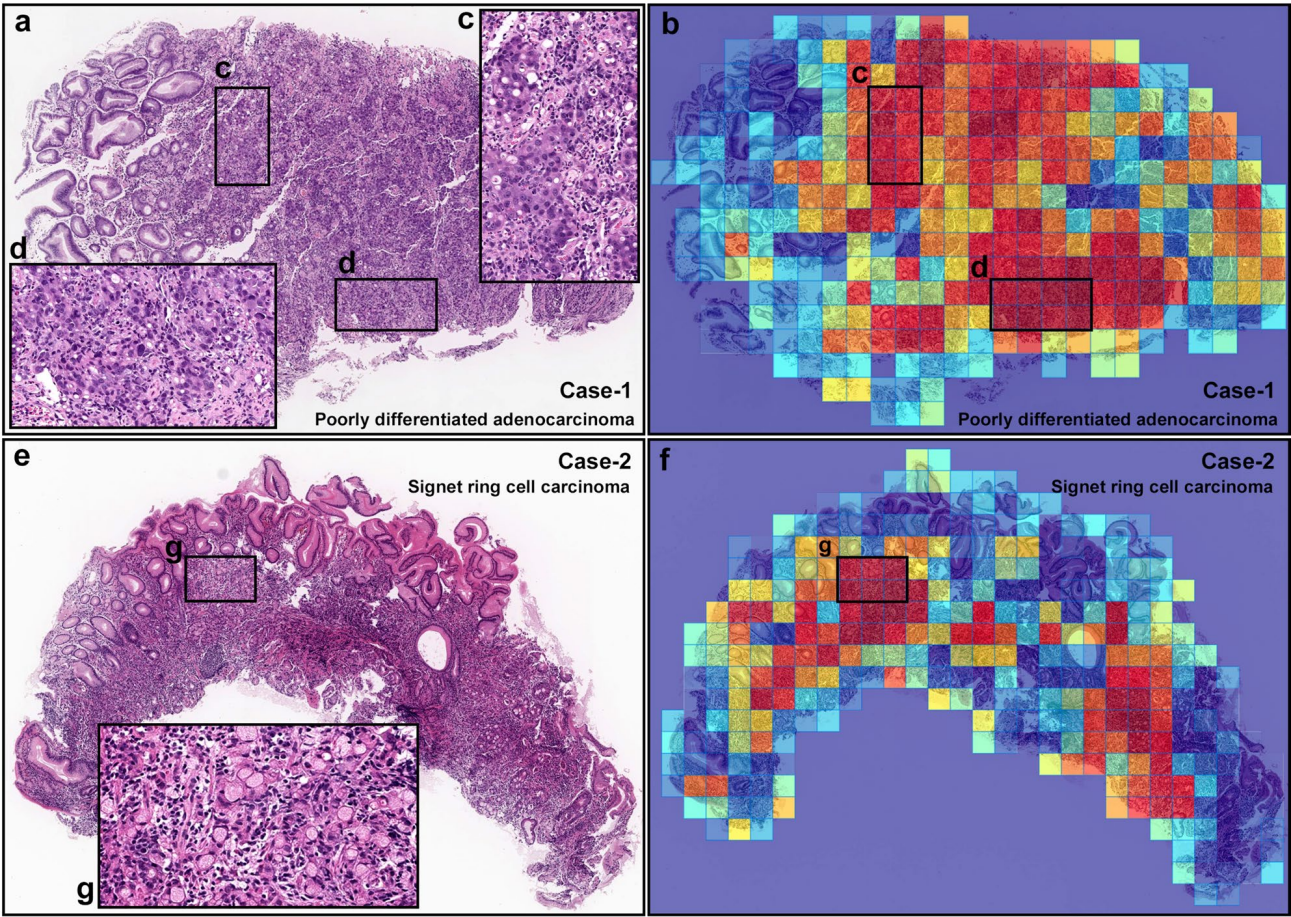
Representative true positive diffuse-type gastric ADC cases from endoscopic biopsy test set. Case-1 (**a**–**d**) is a poorly-differentiated ADC and Case-2 (**e**–**g**) is a SRCC. Heatmap images (**b** and **f**) show true positive predictions of poorly-differentiated ADC (**b**) and SRCC (**f**) cells and they correspond respectively to (**a**) and (**e**) H&E histopathology. The high magnification images (**c**, **d**, and **g**) show representative poorly-differentiated ADC (**c** and **d**) and SRCC (**g**) cellular morphology. Model applied at $\times$ 20, where the 224 $\times$ 224px heatmap square represents 112 $\times$ 112 ${\upmu }$m$^2$.

**Figure 4:**
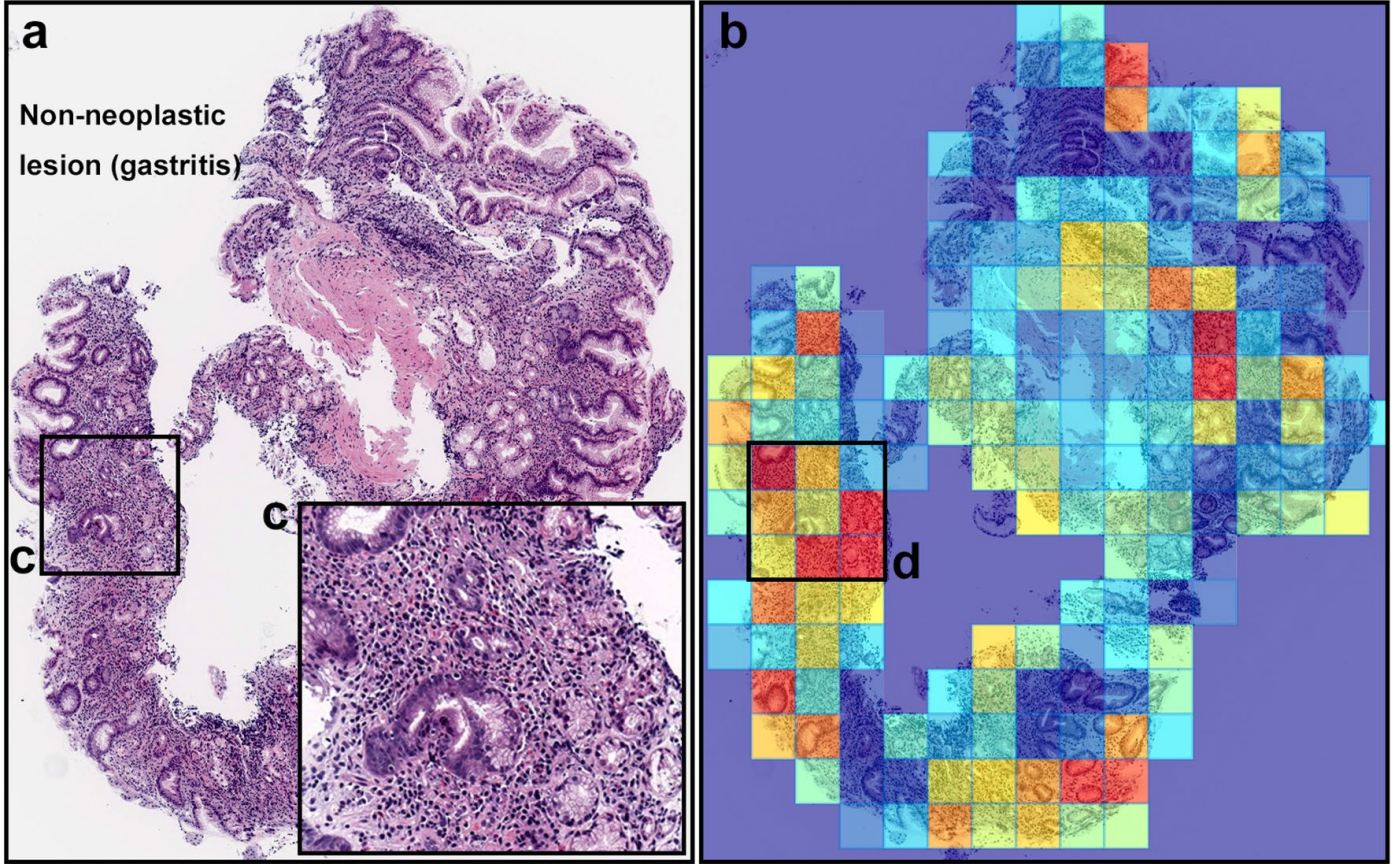
A representative example of diffuse-type ADC false-positive prediction outputs. (**a**) is a non-neoplastic lesion (chronic gastritis). Heatmap images (**b**) exhibited false positive predictions of diffuse-type ADC. The inflammatory tissue with plasma cell infiltration (**c**) is the possible main cause of false positive (**d**) due to its analogous nuclear and cellular morphology to diffuse-type ADC cells. Model applied at $\times$ 20, where the 224 $\times$ 224px heatmap square represents 112 $\times$ 112 ${\upmu }$m$^2$.

**Figure 5:**
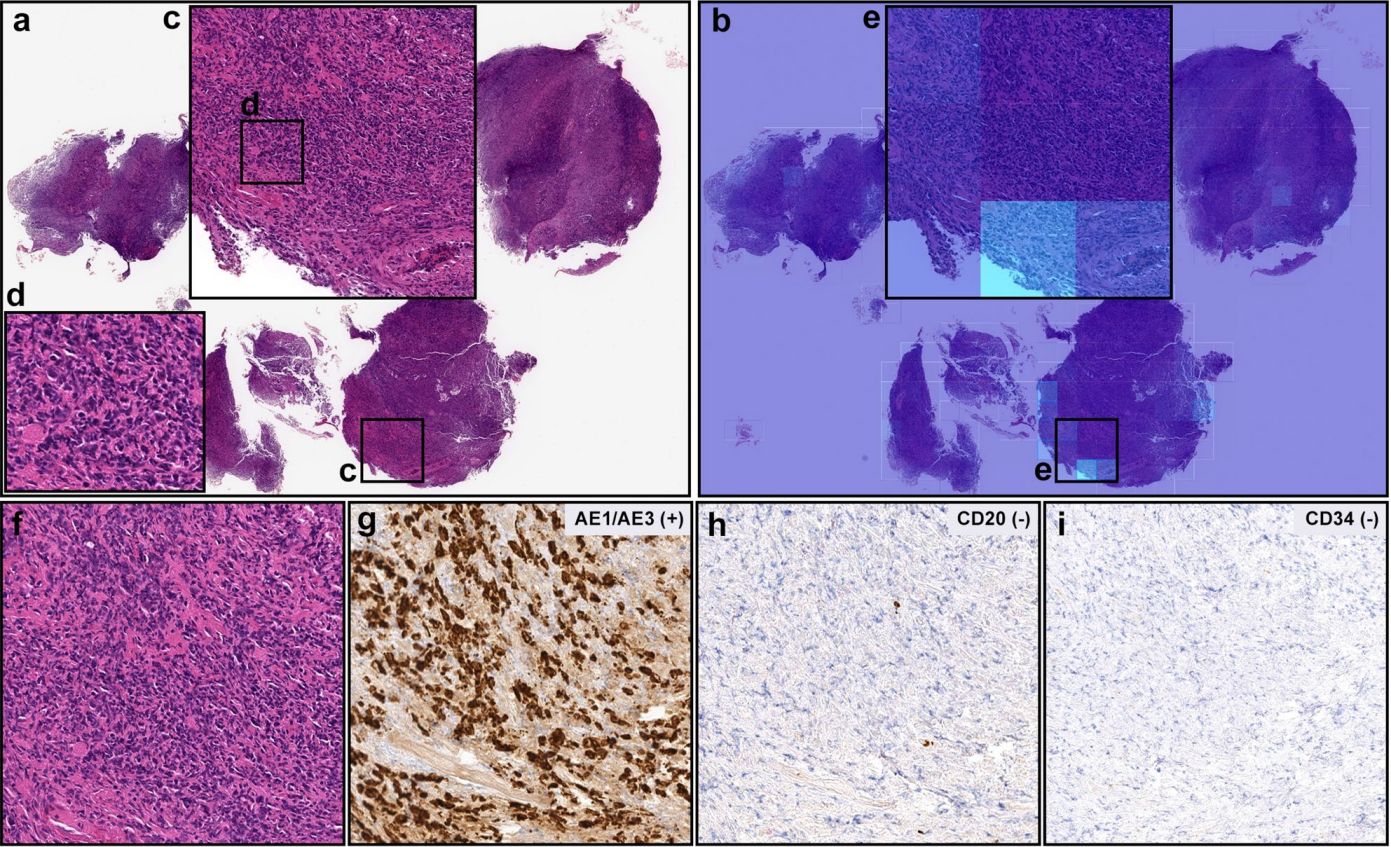
A representative false negative case. In (**a**), there are numerous number of infiltrating degenerative cancer cells (**c**, **d**, **f**) which were not predicted as diffuse-type ADC cells on heatmap image (**b**, **e**) in necrotic and granulation tissues. After immunohistochemical stainings with AE1/AE3 (**g**), CD20 (**h**), and CD34 (**i**), infiltrating cancer cells (**f**) exhibited AE1/AE3 positive, CD20 negative, and CD34 negative, indicating cancer of epithelial origin (carcinoma). Therefore, histopathologically, this case was diagnosed as a diffuse-type differentiated ADC. Model applied at $\times$ 20, where the 224 $\times$ 224px heatmap square represents 112 $\times$ 112 ${\upmu }$m$^2$.

We evaluated our models on five test sets consisting of biopsy specimens originating each from a distinct hospital. Table 3 breaks down the distribution of the WSIs in each test set. For each test set, we computed the ROC AUC for the WSI classification of diffuse-type ADC as well as the log loss, and we have summarised the results in Tables 1, 2 and Fig. 2. Figures 3, 4, and 5 show true positive, false positive, and false negative example heatmap outputs, respectively.

**Table 1:** ROC AUC and log loss results for the four different models as evaluated on the five different test sets.

Method	Source	ROC AUC	Log loss
two-stage $\times$ 10 224	Hospital 1	0.9633 [0.9335, 0.9864]	0.2892 [0.2251, 0.3396]
	Hospital 2	0.9590 [0.9365, 0.9771]	0.1931 [0.1413, 0.2461]
	Hospital 3	0.9903 [0.9771, 0.9983]	0.0651 [0.0435, 0.0948]
	Hospital 4	0.9669 [0.9209, 0.9972]	0.3075 [0.2121, 0.4048]
	Hospital 5	0.9932 [0.9863, 0.999]	0.1249 [0.0914, 0.1511]
one-stage $\times$ 20 512	Hospital 1	0.9835 [0.9776, 0.9973]	0.6243 [0.5222, 0.7274]
	Hospital 2	0.9696 [0.9375, 0.990]	0.3333 [0.2731, 0.400]
	Hospital 3	0.9862 [0.9824, 0.999]	0.3850 [0.3294, 0.4484]
	Hospital 4	0.9774 [0.9369, 0.9968]	1.1246 [0.9182, 1.3698]
	Hospital 5	0.9847 [0.9701, 0.9986]	0.5789 [0.512, 0.662]
one-stage $\times$ 20 224	Hospital 1	0.9594 [0.9314, 0.9818]	0.5874 [0.5324, 0.649]
	Hospital 2	0.9759 [0.9508, 0.993]	0.1988 [0.1810, 0.2202]
	Hospital 3	0.9751 [0.9464, 0.9944]	0.4177 [0.3942, 0.4501]
	Hospital 4	0.9714 [0.9281, 0.9935]	0.9354 [0.8142, 1.0714]
	Hospital 5	0.9774 [0.9498, 0.9978]	0.4428 [0.4128, 0.4682]
one-stage $\times$ 10 224	Hospital 1	0.8989 [0.8278, 0.9473]	1.4383 [1.2866, 1.6029]
	Hospital 2	0.9118 [0.8712, 0.9476]	0.2699 [0.2242, 0.3253]
	Hospital 3	0.8685 [0.7532, 0.9543]	1.0267 [0.9322, 1.1403]
	Hospital 4	0.8293 [0.6485, 0.9601]	1.8348 [1.5575, 2.1335]
	Hospital 5	0.9137 [0.8440, 0.9735]	1.1132 [1.0001, 1.2146]

First row group corresponds to the two-stage model, and the remaining three correspond to the one-stage models, with variations in magnification and tile size. Confidence intervals are between brackets.

**Table 2:** Accuracy, sensitivity, and specificity for the five test sets using the model at $\times$ 20 with tile size 512 $\times$ 512px.

	Accuracy	Sensitivity	Specificity
Hospital 1	0.924 [0.899–0.952]	0.961 [0.911–1.000]	0.921 [0.892–0.951]
Hospital 2	0.946 [0.924–0.963]	0.907 [0.821–0.979]	0.950 [0.929–0.969]
Hospital 3	0.925 [0.903–0.949]	0.911 [0.862–0.962]	0.926 [0.901–0.949]
Hospital 4	0.888 [0.818–0.930]	0.903 [0.851–0.953]	0.887 [0.815–0.931]
Hospital 5	0.903 [0.875–0.929]	0.999 [0.955–1.000]	0.899 [0.871–0.926]

All these values were computed at a probability threshold of 0.5. The ROC curves in Fig. 2 provide more representative values for sensitivity and specificity across the range of threshold from 0.0 to 1.0.

### Evaluation on surgical and frozen sections

**Figure 6:**
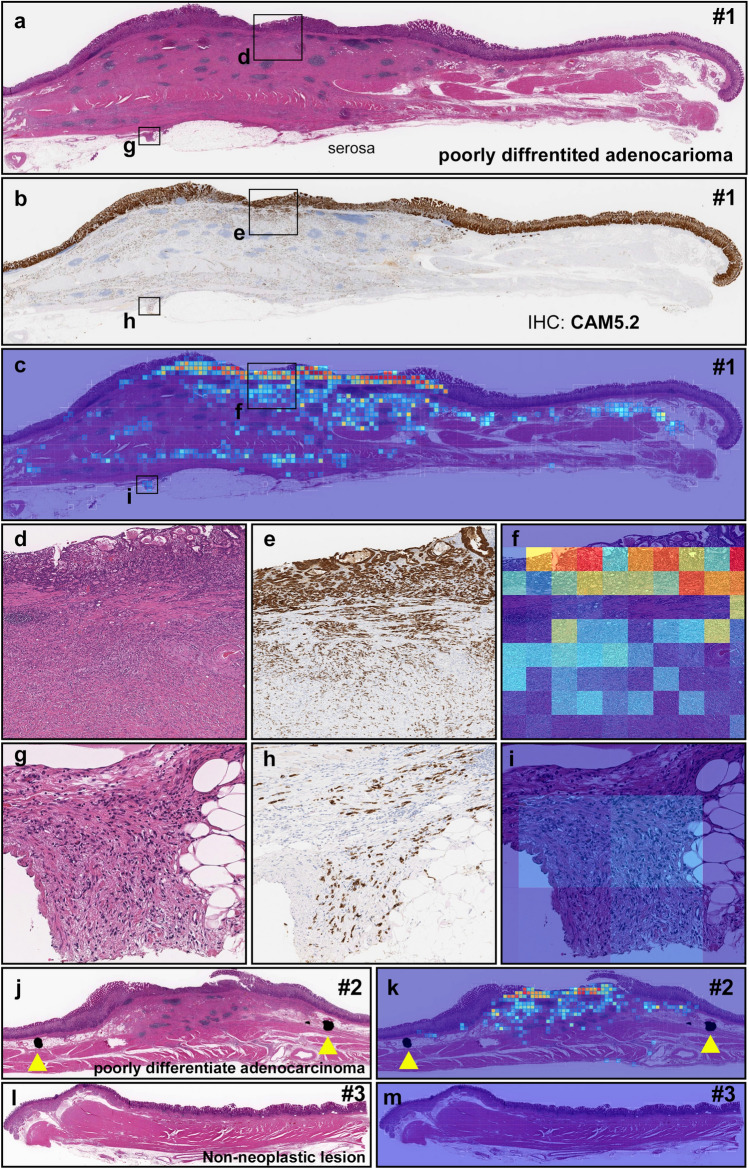
A representative surgically resected case serial specimens for diffuse-type differentiated ADC. Serial specimens: #1 (**a**–**i**), #2 (**j**, **k**), and #3 (**l**, **m**). In #1 (**a**), diffuse-type differentiated ADC cells which are positive for CAM5.2 (**b**, **e**, **f**) invaded from submucosa (**d**, **e**) to subserosa (**g**, **h**). (**c**, **f**, **i**) show true positive probability heatmaps for invading diffuse-type differentiated ADC. In #2 (**j**), true positive probability heatmap image for invading diffuse-type differentiated ADC (**k**) cell invading area was corresponded to surgical pathologists marked area with ink-dots (yellow-triangles) (**j**). #3 (**l**) is a non-neoplastic tissue without any sign of cancer cell invading which is corresponded to the heatmap image (**m**). Model applied at $\times$ 20, where the 224 $\times$ 224px heatmap square represents 112 $\times$ 112 ${\upmu }$m$^2$.

**Figure 7:**
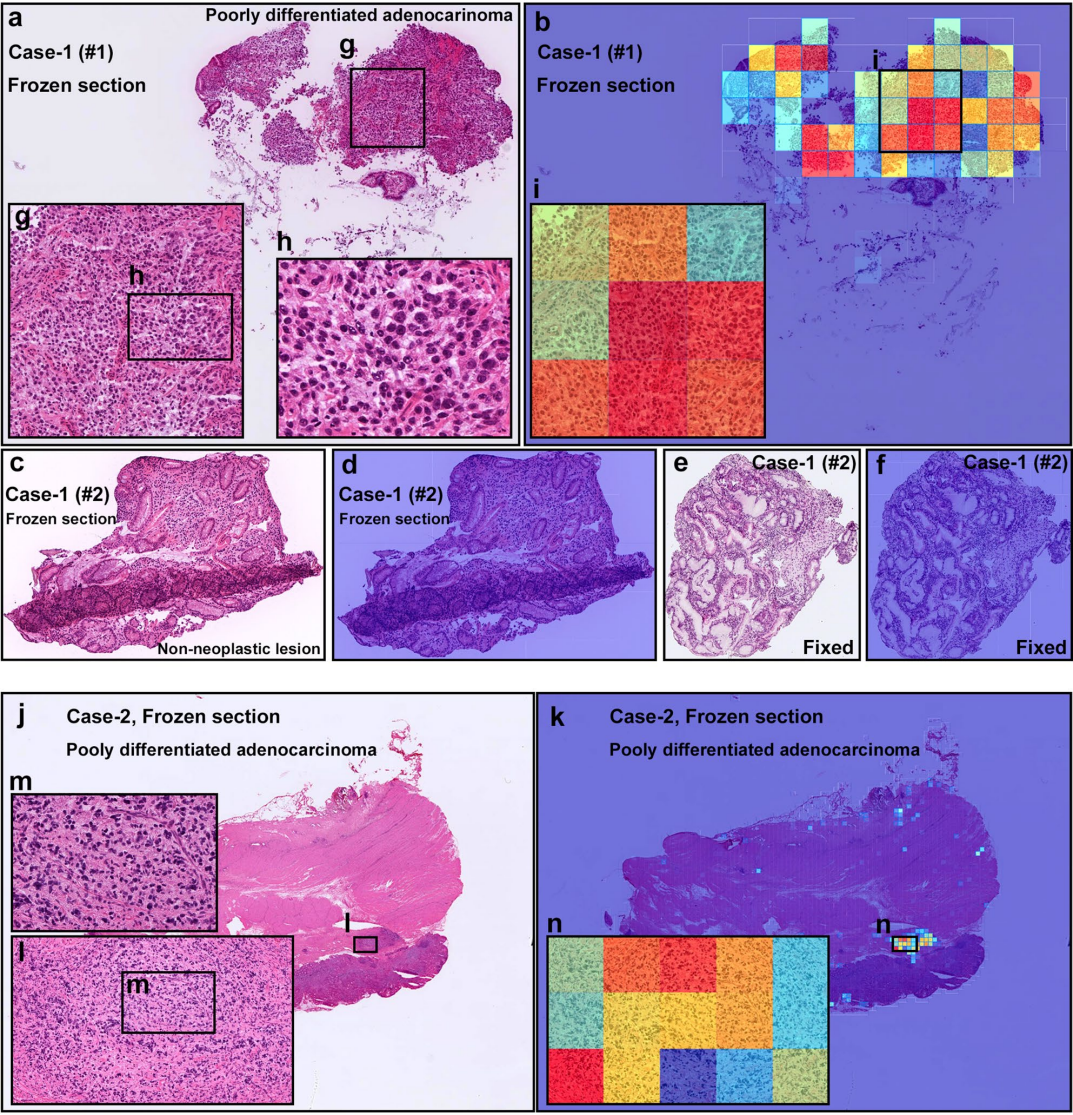
Representative two cases of frozen section specimens for diffuse-type differentiated ADC. Case-1 consisted of two specimens (#1 and #2). In Case-1 (#1) (**a**), the heatmap image (**b**) shows true positive predictions of diffuse-type differentiated ADC cells (**g**–**i**). In Case-1 (#2) frozen section specimen (**c**), there was no cancer cells indicating non-neoplastic specimen which was corresponded to the heatmap image (**d**). The frozen section (#2) was double-checked after conventional fixation (**e**). No cancer cells were observed in the fixed specimen as well (**e**) which was corresponded to the heatmap image (**f**). In Case-2 (**j**), the heatmap image (**k**) shows true positive predictions of diffuse-type differentiated ADC cells (**l**–**n**). Model applied at $\times$ 20, where the 224 $\times$ 224px heatmap square represents 112 $\times$ 112 ${\upmu }$m$^2$.

In addition to the biopsy samples, we have applied the model on the small number of surgical and frozen sections. Figures 6 and 7 show example output predictions on such cases. We see the model was capable of detection diffuse-type ADC on such sections.

## Discussion

In this work, we trained models for the classification of gastric diffuse-type ADC from biopsy WSIs. We used the partial transfer learning approach with a hard mining of false positives to train the models on a dataset obtained from a single hospital, and we evaluated them on five different test sets originating from different hospitals. Overall, we obtained high ROC AUCs in the range of 0.95–0.99.

The best performing models were the one-stage model at $\times$ 20 magnification and 512 $\times$ 512px tile size and the 2-stage model at $\times$ 10 magnification and 224 $\times$ 224px tile size. For the one-stage model, training at $\times$ 20 magnification led to an increase in performance, where the average ROC AUC increased from 0.87 to 0.97 for the five test sets. The increase in magnification was most likely essential in decreasing the false positive rate. Despite being at $\times$ 10 magnification, the two-stage model still performed well potentially due to having been trained on a much larger datasets (n = 4036) and the use of the RNN model which aims at reducing the false-positives.

The trained model was able to detect well both poorly-differentiated ADC and SRCC cells (see Fig. 3 for an example representative case). The majority of false positives occurred on gastritis cases due to the similarity between diffuse-type ADC and inflammatory cells especially plasma cells (see Fig. 4).

Diffuse-type gastric ADCs composed are composed of diffuse-type cohesive carcinoma and SRCCs^24^, and they show an aggressive biological behavior and poor prognosis^25^. In a previous report, patients with SRCC and diffuse-type differentiated ADC in advanced stages demonstrated significantly lower 10-year overall survival rates than the survival rates of patients with advanced differentiated-type ADCs^26^. The availability of a tool that can aid pathologists in the diagnosis of diffuse-type ADC could potentially accelerate their diagnostic workflow.

## Methods

### Clinical cases and pathological records

For the present retrospective study, a total of 2,929 endoscopic biopsy cases of human gastric epithelial lesions HE (hematoxylin & eosin) stained histopathological specimens were collected from the surgical pathology files of five hospitals: International University of Health and Welfare, Mita Hospital (Tokyo), Kamachi Group Hospitals (Fukuoka), Haradoi Hospital (Fukuoka), and Nishi-Fukuoka Hospital (Fukuoka) after histopathological review of those specimens by surgical pathologists. The experimental protocol was approved by the ethical board of the International University of Health and Welfare (No. 19-Im-007), Kamachi Group Hospitals, Haradoi Hospital, and Nishi-Fukuoka Hospital. All research activities complied with all relevant ethical regulations and were performed in accordance with relevant guidelines and regulations in the all hospitals mentioned above. Informed consent to use histopathological samples and pathological diagnostic reports for research purposes had previously been obtained from all patients prior to the surgical procedures at all hospitals, and the opportunity for refusal to participate in research had been guaranteed by an opt-out manner. The test cases were selected randomly, so the obtained ratios reflected a real clinical scenario as much as possible. All WSIs were scanned at a magnification of $\times$ 20.

### Dataset and annotations

The pathologists excluded cases that were inappropriate or of poor quality for this study. The diagnosis of each WSI was verified by at least two pathologists. Table 3 breaks down the distribution of the datasets into training, validation, and test sets. Hospitals which provided histopathological cases were anonymised (e.g., Hospital 1–5). The training and test sets were solely composed of WSIs of endoscopic biopsy specimens. The patients’ pathological records were used to extract the WSIs’ pathological diagnoses. 353 WSIs from the training and validation sets had a diffuse-type ADC diagnosis. They were manually annotated by a group of two surgical pathologists who perform routine histopathological diagnoses. The pathologists carried out detailed cellular-level annotations by free-hand drawing around diffuse-type ADC cells that corresponded to poorly-differentiated ADC or SRCC. The other ADC (n = 571) and non-neoplastic subsets (n = 1116) of the training and validation sets were not annotated and the entire tissue areas within the WSIs were used. Each annotated WSI was observed by at least two pathologists, with the final checking and verification performed by a senior pathologist.

**Table 3:** Distribution of WSIs in the training, validation, and test sets.

Set	Source	Diffuse-type ADC	Other ADC	Non-neoplastic	Total
2-stage: train	Hospital 2	333	541	–	874
2-stage: validation	Hospital 2	20	30	–	50
1-stage: train	Hospital 2	333	541	1076	1950
1-stage: validation	Hospital 2	20	30	40	90
Test	Hospital 1	25	96	234	355
	Hospital 2	54	55	407	516
	Hospital 3	10	12	473	495
	Hospital 4	10	20	113	143
	Hospital 5	19	38	439	496

### Deep learning models

For the detection of diffuse-type ADC, we used two approaches: one-stage and two-stage. The one-stage approach consisted in training the CNN as a binary classifier to directly classify diffuse-type ADC. The two-stage approach consisted in combining the output from an existing model^21^ that differentiates between ADC, adenoma, and non-neoplastic^21^, followed by a model trained to differentiate between diffuse-type ADC and other ADC. We trained all the models using the partial fine-tuning approach^23^. This method simply consists in using the weights of an existing pre-trained model and only fine-tuning the affine parameters of the batch normalisation layers and the final classification layer. We have used the EfficientNetB1^27^ model starting with pre-trained weights on ImageNet. The total number of trainable parameters was only 63,329.

To apply the CNN on the WSIs, we performed slide tiling by extracting square tiles from tissue regions. On a given WSI, we detected the tissue regions and eliminated most of the white background by performing a thresholding on a grayscale version of the WSI using Otsu’s method^28^. During prediction, we perform the tiling in a sliding window fashion, using a fixed-size stride, to obtain predictions for all the tissue regions. During training, we initially performed random balanced sampling of tiles from the tissue regions, where we tried to maintain an equal balance of each label in the training batch. To do so, we placed the WSIs in a shuffled queue such that we looped over the labels in succession (i.e. we alternated between picking a WSI with a positive label and a negative label). Once a WSI was selected, we randomly sampled $\frac{\text {batch size}}{\text {num labels}}$ tiles from each WSI to form a balanced batch. To maintain the balance on the WSI, we over-sampled from the WSIs to ensure the model trains on tiles from all of the WSIs in each epoch. We then switched into hard mining of tiles once there was no longer any improvement on the validation set after two epochs. To perform the hard mining, we alternated between training and inference. During inference, the CNN was applied in a sliding window fashion on all of the tissue regions in the WSI, and we then selected the *k* tiles with the highest probability for being positive if the WSI was negative and the *k* tiles with the lowest probability for being positive if the WSI was positive. This step effectively selects the hard examples which the model is struggling with. The selected tiles were placed in a training subset, and once that subset contained *N* tiles, the training was run. This method is similar to the weakly supervised training method as described by Kanavati et al.^29^. We used $k = 16$, $N=256$, and a batch size of 32.

From the WSIs with diffuse-type ADC, we sampled tiles based on the free-hand annotations. If the WSI contained annotations for cancer cells, then we only sampled tiles from the annotated regions as follows: if the annotation was smaller than the tile size, then we sampled the tile at the centre of the annotation regions; otherwise, if the annotation was larger than the tile size, then we subdivided the annotated regions into overlapping grids and sampled tiles. Most of the annotations were smaller than the tile size. On the other hand, if the WSI did not contain diffuse-type ADC, then we freely sampled from the entire tissue regions.

The first stage model^21^ is based on the InceptionV3 architecture^30^ is followed by a single layer recurrent neural network. It was trained with an input tile size of $512\times 512$ px on WSIs with a magnification of $\times$ 10. As the 2nd stage model was only trained on ADC, we used the product of the probability outputs to compute the probability that a given WSI has diffuse-type ADC:

$$\begin{aligned} P({\text {diffuse-type ADC}}) = P_2({\text {diffuse-type ADC}}| {\text {ADC}}) \times P_1({\text {ADC}}), \end{aligned}$$

where $P_1(\text {ADC})$ is the probability output from the 1st stage model and $P_2(\text {diffuse-type ADC}| \text {ADC})$ is the probability from the 2nd stage model.

To perform inference on the WSI (i.e. obtain a WSI prediction), we applied the model in a sliding window fashion on all the tissue regions, and we then took the maximum probability of the tiles and used that as the WSI probability.

We trained the models with the Adam optimisation algorithm^31^ with the following parameters: $beta_1=0.9$, $beta_2=0.999$, and a batch size of 32. We used a starting learning rate of 0.001 when training the model from scratch, and 0.0001 when fine-tuning. We applied a learning rate decay of 0.95 every 2 epochs. We used the categorical cross entropy loss function. We used early stopping by tracking the performance of the model on a validation set, and training was stopped automatically when there was no further improvement on the validation loss for 10 epochs. The model with the lowest validation loss was chosen as the final model.

### Software, hardware, and statistical analysis

We implemented the models using TensorFlow^32^. We calculated the AUCs in python using the scikit-learn package^33^ and performed the plotting using matplotlib^34^. We performed image processing, such as the thresholding with scikit-image^35^. We computed the 95% CIs estimates using the bootstrap method^36^ with 1000 iterations. We used openslide^37^ to perform real-time slide tiling. We trained the models on a single g4dn.2xlarge instance on amazon AWS which has an NVIDIA T4 Tensor Core GPU, 8 CPUs, and 32GB of RAM.

## Data Availability

The data that support the findings of this study are available from International University of Health and Welfare, Mita Hospital (Tokyo), Kamachi Group Hospitals (Fukuoka), Haradoi Hospital (Fukuoka), and Nishi-Fukuoka Hospital (Fukuoka), but restrictions apply to the availability of these data, which were used under a data use agreement which was made according to the Ethical Guidelines for Medical and Health Research Involving Human Subjects as set by the Japanese Ministry of Health, Labour and Welfare, and so are not publicly available. However, the data are available from the authors upon reasonable request for private viewing and with permission from the corresponding five medical institutions within the terms of the data use agreement and if compliant with the ethical and legal requirements as stipulated by the Japanese Ministry of Health, Labour and Welfare. Access to the data can also be obtained by entering into a similar data sharing agreement with the medical institutions.

## Code availability

To train the classification model in this study we used the publicly available TensorFlow training script available at https://github.com/tensorflow/models/tree/master/official/vision/image_classification.
